# An Efficient Location Verification Scheme for Static Wireless Sensor Networks

**DOI:** 10.3390/s17020225

**Published:** 2017-01-24

**Authors:** In-hwan Kim, Bo-sung Kim, JooSeok Song

**Affiliations:** Department of Computer Science, College of Engineering, Yonsei University, 50 Yonsei-ro, Seodaemun-gu, Seoul 03722, Korea; bokor@yonsei.ac.kr (B.K.); jssong2526@yonsei.ac.kr (J.S.)

**Keywords:** sensor, location verification, static wireless sensor networks (WSNs)

## Abstract

In wireless sensor networks (WSNs), the accuracy of location information is vital to support many interesting applications. Unfortunately, sensors have difficulty in estimating their location when malicious sensors attack the location estimation process. Even though secure localization schemes have been proposed to protect location estimation process from attacks, they are not enough to eliminate the wrong location estimations in some situations. The location verification can be the solution to the situations or be the second-line defense. The problem of most of the location verifications is the explicit involvement of many sensors in the verification process and requirements, such as special hardware, a dedicated verifier and the trusted third party, which causes more communication and computation overhead. In this paper, we propose an efficient location verification scheme for static WSN called mutually-shared region-based location verification (MSRLV), which reduces those overheads by utilizing the implicit involvement of sensors and eliminating several requirements. In order to achieve this, we use the mutually-shared region between location claimant and verifier for the location verification. The analysis shows that MSRLV reduces communication overhead by 77% and computation overhead by 92% on average, when compared with the other location verification schemes, in a single sensor verification. In addition, simulation results for the verification of the whole network show that MSRLV can detect the malicious sensors by over 90% when sensors in the network have five or more neighbors.

## 1. Introduction

**Figure 1:**
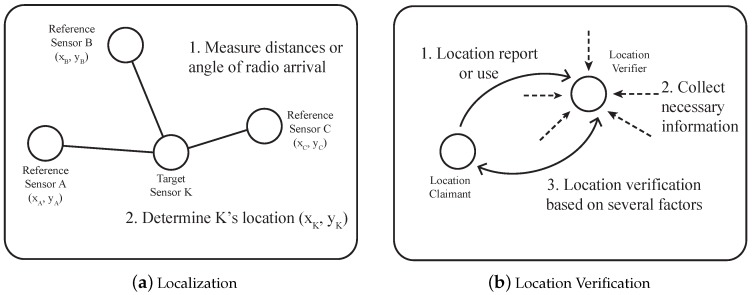
Localization and location verification.

Wireless sensor networks (WSNs) have been one of the most popular topics for many years. The networks consist of a large number of low-cost sensors with wireless communication interfaces and enable many applications, such as environment monitoring, target tracking or rescuing. In those applications, location information plays an important role to make the sensed data more meaningful. Furthermore, the delivery of the data relies on the position of sensors when geographical routing is used as the underlying routing protocol. To enable those applications, sensors usually get their positions by the location estimation process called localization. One of the famous localization methods is Global Positioning Systems (GPS), but other schemes, such as magnetic positioning, dead reckoning and data-fusion-based positioning, are also possible. For instance, anchor sensors who are equipped with video and a compass can be used to estimate the location when GPS is not available [1]. Since many of the localization processes are designed in a benign environment where no attackers exist, it may not be possible to provide the correct location with any attacker. Thus, the location estimation process known as secure localization has been proposed to protect the localization by utilizing hop-distance information, game-theory or trust models [2,3,4,5,6,7,8,9]. Unfortunately, they are not enough to eliminate the wrong location estimations in some situations, e.g., reference sensors that provide the absolute coordinate are compromised or sensors report a false location by themselves. Hence, the location verification process that checks the reported location of sensors becomes necessary. Figure 1 shows the general concept of the localization and location verification. As shown in Figure 1a, localization generally measures the distances or angles of radio arrival between a set of reference sensors who know their location and the target sensor who wants to know its location. Based on the measurements, the location of the target sensor is determined. Secure localization uses some physical constraints, such as distance inconsistency between sensors, to prevent any malicious sensors from cheating in the location estimation process. In the case of location verification, it usually occurs when the acquired location is used in an application. For example, if the location of a sensor is suspicious, a sensor that is called the verifier collects the necessary information and performs location verification as shown in Figure 1b. This location verification problem can be formulated in many different mathematical ways by reflecting the characteristics of the algorithms [10,11,12,13,14,15,16,17,18,19,20,21].

Location verification schemes in WSNs can be categorized as on-spot and in-region verification [15]. On-spot verification is to verify whether the true location of a sensor is the same as its claimed location, and in-region verification is to verify whether a sensor is within a certain region. In each category, several location verification schemes have been proposed to check the reported location of sensors with special requirements [10,11,12,13,14,15,16,17,18,19,20,21]. The special requirements, such as special hardware, the active participation of neighbors, a dedicated verifier and a trusted third party, impose a burden on the sensors in terms of hardware cost, communication overhead and computation overhead.

In this paper, we propose an efficient location verification scheme in static WSNs, which falls into the on-spot verification. To eliminate any special requirements that cause communication and computation overhead, the proposed scheme utilizes the common knowledge between claimant and verifier: one claiming its claimed location and the other trying to verify the location, respectively. The common knowledge is called the MSR (mutually-shared region) token and should be some information that can be derived exclusively between claimant and verifier. In other words, it works in a peer-to-peer fashion with implicit help from the common neighbors of the claimant and verifier. For example, sensors collect the sensed data coming from neighbors for a certain amount of time and use relevant data to verify the location of the claimant after the appropriate calculation.

The advantage of the proposed scheme is that it can effectively verify the claimed location of a sensor without incurring significant communication and computation overhead. This is because our scheme does not request the participation of all of the sensors in the network and does make use of simple calculations for the location verification. In addition, there are no requirements of special hardware, dedicated verifiers and a trusted third-party for the verification. The contributions of this work can be described as follows:

- No prearranged setup or sensor deployment knowledge is required. The proposed scheme works based on the MSR token, which will be explained in Section 4. The MSR token can be acquired independently by the claimant and verifier without explicitly exchanging of information.
- The proposed scheme works in a localized way, not involving the entire networks whenever location verification is needed. Unlike the other location verification schemes, the proposed scheme can perform the verification process with sensors around the area where the location verification is required. Many of the other location verification schemes involve the entire network for the verification process even when only one sensor has a problem with its location.
- Little communication and computation overhead will be caused by the location verification scheme. The scheme utilizes the data packets, such as temperature sensing data from sensors, to deliver the information for location verification. With this implicit data exchange, two verification packets are needed to verify a single location claim. Moreover, simple calculation in the verification algorithm does not incur much computation overhead for the sensors.

The remainder of this paper is organized as follows. Section 2 discusses the related works. Section 3 provides the system and attack model for the proposed scheme. Section 4 describes the proposed scheme, which is named mutually-shared region-based location verification (MSRLV). Afterward, the security and performance analysis are provided in Section 5, and the verification performance of the proposed scheme is evaluated in Section 6. Section 7 will discuss further considerations on the performance improvement. Finally, Section 8 concludes the paper.

## 2. Related Works

There have been many secure localization schemes [2,3,4,5,6,7,8] to give honest sensors the capability of securely positioning themselves even in the presence of attackers. Even though the secure localization protects the location estimation process from several attacks, the location estimation can still go wrong in some situations, e.g., compromised sensors report a false location intentionally. Therefore, many researchers have worked on the location verification in WSNs [10,11,12,13,14,15,16,17,18,19,20,21]. Location verification schemes can be categorized as in-region and on-spot verification [15]. In-region verification usually measures the round-trip time of challenge-response packets to calculate the distance between two sensors and uses the distance to determine whether a sensor is located inside some region. This can be done with the distance-bounding [22], which requires extra hardware to measure the timing with nanosecond precision. The echo protocol proposed by Sastry et al. is an example of addressing the location verification problem based on the distance-bounding [10]. It used radio wave and ultrasound to determine whether a sensor is inside a specific region or not.

On the other hand, on-spot verification usually verifies whether the claimed location of the sensors is correct [11,12,13,14,15,16,17,18]. This type of verification mainly uses pre-knowledge of the sensor deployment, the hop-distance relationship, graph theory or neighborhoods’ observation to verify the claimed location of sensors. The following research works are the examples of on-spot verification.

Du et al. proposed the location anomaly detection (LAD) scheme as the second-line of defense for the localization [12]. In their paper, they formulated the localization anomaly problem as an anomaly intrusion detection problem and proposed the detection method. The method is based on the consistency between estimated location and the deployment knowledge of sensors. The scheme needs to collect packets from all sensors for location verification to generate several matrices reflecting the observations on other sensors. It is effective in detecting attacks against the localization, but requires prior sensor deployment knowledge and involvement of many sensors for the location verification.

Ekici et al. proposed the on-spot verification scheme called Probabilistic Location Verification (PLV). The idea is to utilize the hop-distance between sensors [13]. The hop-distance is a statistical relationship between the hop count and the Euclidean distance the broadcast packet traverses. In this scheme, a small number of verifiers performs the probability calculation and sends it to the central node. The central node finally calculates the plausibility of the reported location of a sensor to detect the malicious sensors. With the help of probability and statistics, the scheme does not need special hardware, but it still has room to be attacked due to the use of dedicated verifiers and the trusted third party.

Phantom node detection was proposed by Hwang et al. [14]. In phantom node detection, each sensor generates the local map, which is constructed based on the distances among its neighbors to perform the location verification process. Since the process only uses relative distances among neighbors and does not reveal the location of sensors, attackers cannot make their fake location consistent with honest sensors in the local map. In other words, the scheme works based on the assumption that all of the location information of sensors is hidden. The advantage of the scheme is that it can filter out most of the attackers even when they overwhelm honest sensors, and it works in a distributed way. However, it requires all neighbors’ estimation and the broadcast of their mutual distances with much communication between them. Moreover, it could not cover the practical environment where attackers with their known position cooperate to find out the location of honest sensors through any localization method, which consequently makes the phantom detection scheme ineffective.

Later, Wei et al. presented the location verification algorithm that depends on neighborhoods’ observation [15]. The scheme is lightweight because it eliminates the burden of sensors by using a powerful central server to process the location verification. In addition, it does not use special hardware and sensor deployment knowledge. However, it requires the active participation of neighbors and the trusted third party to perform the location verification. Especially, the use of the central server may cause a single point of failure. The centralized approach makes it necessary that too many sensors have to be involved in the location verification process even when a small number of sensors has a problem with their location.

There were more recent research works trying to eliminate the fixed anchors or to detect the unreliable anchors and sensors [16,17,18]. Perazzo et al. suggested the location verification based on verifiable multilateration (VM) [11] in [16]. They used a drone to replace the fixed anchors. Their eventual algorithm focused on designing the path planning algorithm of the drone to do the task of fixed anchors instead. With the algorithm, it shows the possibility of drone-based VM, but they still depend on the fixed anchors.

Miao et al. provided the collaborative localization and location verification to detect the unreliable sensors [17]. They used the virtual force model to calculate the more accurate location of the sensors. Sensors exert the virtual force on each other based on their measured and estimated distances. Based on the force model, they find out the movement of anchors and normal sensors. It works in a distributed manner to eliminate the single point of failure. Even though they have an issue of anchor compromise, this depends on the anchors to initiate the whole process.

Miao et al. developed the distributed location verification by using the reputation system based on the mutual distance inconsistency between sensors [18]. This scheme calculates the reputation of neighbors based on the distances. The distances are estimated by measuring the received signal strength (RSS) of sensors and performing the trilateration. They can detect the movement of sensors by using the reputation result in a distributed manner. However, this still requires fixed anchors.

Even though it is not designed only for WSNs, there are the other approaches of Jerry T. Chiang and Shihao Yan [19,20,21]. They formulated the location verification problem as a kind of detection system, and the location verification is achieved by finding the optimal solution for the formulated problem. This approach helps the system designer to analyze and prove the suggested algorithm in a mathematical way. Those location verification schemes also use the fixed anchors or base stations to find the malicious sensor.

To summarize, the previous research works mandate the active participation of the entire network or neighbors, the use of a dedicated verifier or a trusted third party for the location verification. The proposed scheme, which is categorized as on-spot verification, tries to reduce the communication and computation overhead caused by the active involvement of the entire network or neighbors and the complex calculation procedure. Furthermore, it decreases the sensor deployment cost by eliminating the use of a dedicated verifier or a trusted third party.

## 3. System and Attack Models

In this section, we show the system and attack model for the proposed scheme. The system model describes the network environment, which includes the role and capability of sensors, network functionality and packet’s contents. The attack model depicts the purposes and capabilities of the attackers.

### 3.1. System Model

It is assumed that the networks consist of homogeneous sensors. The sensors are assumed to be static and have the same capability in terms of transmission range, computation power and storage. Assumptions for the network are as follows:

- The network consists of *N* sensors, and the *i*-th sensor is defined as $n_{i}$, where i=1,2,...,N.
- The participants in the proposed location verification can play one of the following roles, i.e., claimant and verifier. A claimant is a sensor who claims its location, and a verifier is the one who checks the validity of the claimed location.
- The communication range of all sensors is the same and defined as *r*.
- $n_{i}$ emits data packet, $data_{i}=\left\{s_{i},L_{c}^{i},nonce_{i}\right\}$, where $s_{i}$ is sensed data, $L_{c}^{i}$ is the claimed location and $nonce_{i}$ is the random value. $L_{c}^{i}$ and $nonce_{i}$ will be used in the location verification process.
- All sensors are able to get their true location, $L_{true}$, using the localization, and we assume that there is no localization error.
- An honest sensor reports its $L_{c}$, and it is the same as $L_{true}$.
- The network is dense enough, and the location of sensors is determined by a uniform distribution.
- Time synchronization is achieved using any proper technologies, e.g., [23]. This is an essential and basic function that makes the sensed data more meaningful in WSNs.

### 3.2. Attack Model

The purpose of an attacker is to gain any benefit by passing the location verification. For example, the attacker announces false location information of itself to take packets from any targeted sensor when geographical routing is used to deliver data. In the other example of the tracking application, the attacker can report its false location to lie about the target’s movement. To successfully cheat regarding its location information, an attacker can do the following behaviors:

- An attacker can compromise any sensor. The compromised sensor is called a malicious sensor and defined as $m_{i}$, where i=1,2,...,K. The total number of malicious sensors in the entire network is K≪N.
- A malicious sensor, $m_{i}$, can report false location information intentionally by sending a message including its forged location, $fdata_{i}=\left\{s_{i},L_{c}^{i},nonce_{i}\right\}$, where $L_{c}^{i}\neq L_{true}^{i}$.
- The attacker tries to eavesdrop on the communications of sensors to get the necessary information to bypass the location verification.
- The attacker’s ability is constrained to the compromised sensor. This means that the attacker can listen within its communication range, *r*.

## 4. The Proposed Scheme: Mutually-Shared Region-Based Location Verification

MSRLV is the location verification algorithm that utilizes a mutually-shared region token (MSR token). The token can be calculated by legitimate sensors only. MSRLV focuses on the verification process, not the localization process. In order to verify the location claim of a sensor, the proposed scheme requires two participants and the MSR token between them for the location verification. In the following subsections, the MSR token and the detailed scheme will be described.

### 4.1. Mutually Shared Region Token

**Figure 2:**
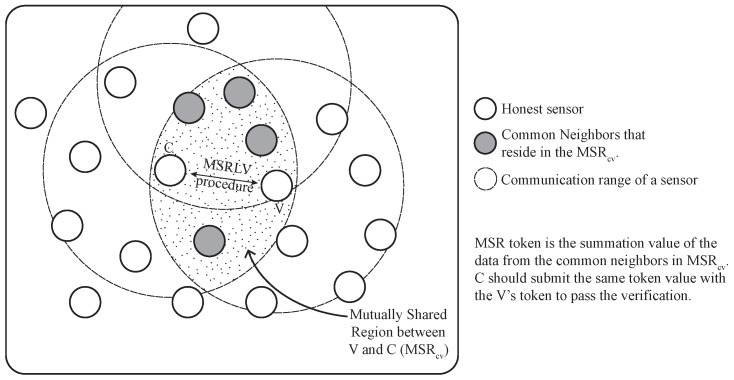
An example of mutually-shared region (MSR) token usage.

The MSRLV makes use of location evidence between the location claimant and verifier, which is called the MSR token. The MSR token is a value that can be derived from the logical or physical features of sensors, but it should not be easy for any third party to guess or calculate it. For example, coordinates, transmission power or packet count from sensors who are involved in location verification cannot be used directly to calculate the token since these are easy to guess. In this paper, the token is the summation of the random values that are coming from common neighbors between two nodes, claimant *c* and verifier *v*, as shown in Figure 2. The token should meet the following properties to provide security of the verification:

- Mutual symmetry: The token should be known to two participants, but unknown to the other sensors. That is, two sensors, verifier *v* and claimant *c*, need to know the same token without explicit message exchange. This implies $msrToken_{v}=msrToken_{c}$, where $msrToken_{i}$ means the MSR token computed at sensor *i*.
- Independent computation: Two participants should be able to compute the token independently. This helps the scheme eliminate the necessity of explicit message exchanges, which might cause vulnerability in the system according to the attack model. This implies $msrToken_{v}=f\left(data_{vc}^{t}\right)=f\left(data_{cv}^{t}\right)=msrToken_{c}$, where f() is the function that is used for the calculation of the token, and $data_{ij}^{t}$ means any kind of data observed at both sensors *i* and *j* and collected by sensor *i* at time *t*.
- Anti-deduction: The token should be safe against any deduction, so it should be processed to prevent adversaries from creating it with a guess. Thus, the use of raw data, such as transmission power, packet count or neighbor count, that is easy to guess or estimate should be avoided. This implies $\left(msrToken_{v}=msrToken_{c}\right)\neq msrToken_{m}$, where *m* means a malicious sensor.

### 4.2. D-Filtering

**Figure 3:**
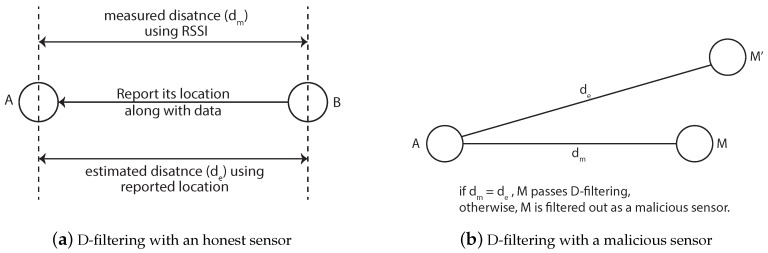
Distance-inconsistency filtering (D-filtering) (**a**) with an honest sensor and (**b**) with a malicious sensor.

The distance-inconsistency filtering (D-filtering) is the process of filtering a sensor who shows an inconsistency in its measured and estimated distances to the other sensor. The measured distance can be acquired by simple distance measuring methods, such as RSS or time-of-flight (ToF), and the estimated distance can be obtained by the calculation based on the reported location information. The filtering is used to perform the simple distance check between two sensors during the proposed location verification process. In this paper, Received Signal Strength Indicator (RSSI) is used to acquire the measured distance. Figure 3 shows the concept of D-filtering.

As show in Figure 3a, a sensor *A* measures the distance to the sensor *B* using RSSI. At the same time, *A* can get the estimated distance to *B* using the reported location of *B*. *A* compares the measured distance to *B* and the estimated one to *B*. If the distances are the same, *B* is considered as a potentially honest sensor, otherwise as a potentially malicious sensor. Figure 3b shows the D-filtering case with a malicious sensor. Since the distances may also be forged by an attacker by falsely reporting its location to the other place while maintaining consistent distances, the sensors who pass the D-filtering are not totally trusted. The detailed analysis of the effect of D-filtering will be provided in Section 5.1.3.

### 4.3. Description of MSRLV Scheme

The MSRLV is triggered when a sensor needs to assure the location of the other sensor. For example, a sensor has to verify the location of the next relay sensor when it uses geographical routing. This is because the geographical routing depends on the location of the sensors to deliver packets successfully. In the other possible scenario, the MSRLV can be triggered when a sensor is noticed for which the received location report from the other sensor is different from its expected location. The expected location of the sensor can be estimated based on the accumulated data from that sensor. In either case, a sensor who triggers the verification acts as a verifier, and the other sensor who claims its location becomes a claimant.

When MSRLV is triggered, the verifier tries to check the legitimacy of a claimant’s location through the D-filtering. The D-filtering is the process of checking the inconsistency of the measured and estimated distances between two sensors. If there is no problem with those distances, the verifier sends a verification request to the claimant. Without this filtering, honest sensors will suffer from verification failure. This will be explained in more detail in the later Section 4.4.

**Figure 4:**
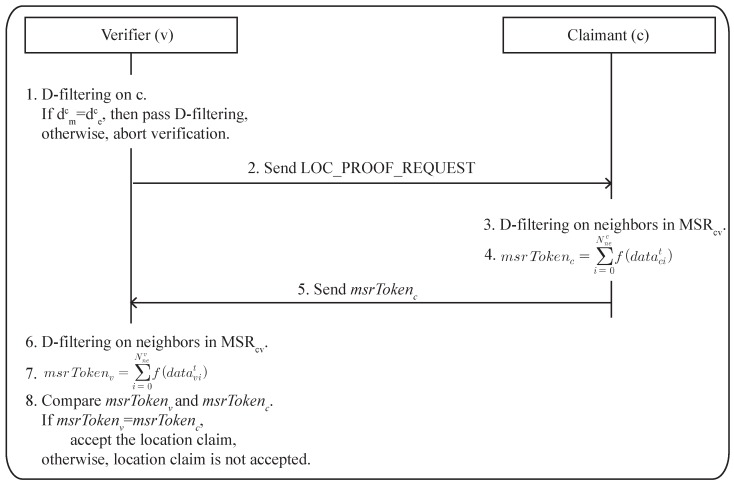
The mutually-shared region-based location verification (MSRLV) process.

The claimant that receives the verification request then calculates the MSR token with the D-filtering on its neighbors. As explained in the previous section, the token should have three properties to guarantee that any other sensors are not able to formulate the same token without being at the correct location. After being calculated, the token is sent to the verifier. Finally, the verifier does the same procedure by itself to calculate the token and then matches them. If they match, the claimed location is verified successfully. Otherwise, it fails. Reactions to the verification failure can be different according to the scenario. For instance, the verification failure may invoke another location verification against a newly-selected relay sensor in the geographical routing case. In the case of temperature measurement application, the data from the unverified sensor may be just removed from the collected dataset, and the sensor can be on the blacklist. The steps of the proposed scheme are shown in Figure 4 and described as follows.

- Step 1. A sensor triggers a location verification process according to the situation mentioned above and becomes a verifier *v*. *v* performs the D-filtering on the claimant *c*. If *c* does not pass the filtering, *v* assumes that *c* is trying to fake its location and terminates the verification process here.
  $$\begin{array}{cc} v: & D-filtering\;on\;c.\\ v: & if\;d_{m}^{c}=d_{e}^{c},\;then\;pass\;D-filtering.\\ & otherwise,\;abort\;the\;verification. \end{array}$$
  where $d_{m}^{c}$ is the measured distance to *c* and $d_{e}^{c}$ is the estimated distance to *c*.
- Step 2. *v* sends a LOC_PROOF_REQUEST packet to *c*. A LOC_PROOF_REQUEST packet asks *c* to send back the MSR token.
  v→c:LOC_PROOF_REQUEST
- Step 3. Upon receiving a LOC_PROOF_REQUEST, *c* performs the D-filtering to filter out the malicious sensors that reside in $MSR_{cv}$.
  $$c:D-filtering\;on\;neighbors\;in\;MSR_{cv}.$$
- Step 4. *c* calculates the MSR token, $msrToken_{c}$, based on the data that it has collected from neighbors in $MSR_{cv}$, within the last *t* seconds. $msrToken_{c}$ can be calculated as follows:
  (1) $$c:msrToken_{c}=\sum_{i=1}^{N_{ne}^{c}}f\left(data_{ci}^{t}\right)\;$$
  where $data_{ci}^{t}$ is the neighbor *i*’s newest data collected by *c* within the last *t* seconds, $N_{ne}^{c}$ is the number of neighbors of *c* and f() is the filtering function that is used to select the common neighbors in $MSR_{cv}$ from the neighbor table.
- Step 5. *c* sends the calculated $msrToken_{c}$ to *v*.
  $$c\to v:msrToken_{c}$$
- Step 6 to 8. *v* confirms whether the received $msrToken_{c}$ matches with the $msrToken_{v}$. A $msrToken_{v}$ is calculated by *v* in the same way as in Steps 3 and 4. If it matches, *v* accepts the location claim from *c*. Otherwise, the location claim is not accepted.
  (2) $$\begin{array}{cc} v: & D-filtering\;on\;neighbors\;in\;MSR_{cv}.\\ v: & msrToken_{v}=\sum_{i=1}^{N_{ne}^{v}}f\left(data_{vi}^{t}\right)\;\\ v: & if\;msrToken_{c}==msrToken_{v},\;accept\;the\;location\;claim.\\ & otherwise,\;location\;claim\;is\;not\;accepted. \end{array}$$

The MSR token calculation process works based on the following assumption: the neighbor table is constructed without an error. For this, we assume that the time synchronization is already done, and the exchange of the data between neighbors is achieved through the underlying network protocols. Thus, any failure while calculating the MSR token is interpreted as the attempt of a malicious sensor to bypass the verification.

**Figure 5:**
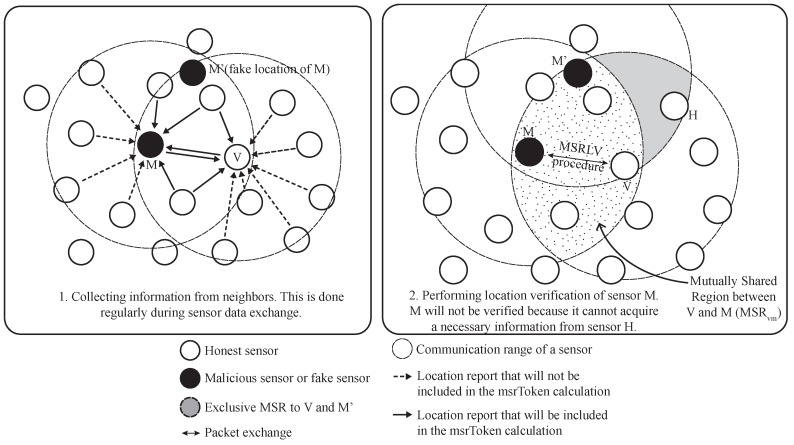
An example of detecting a malicious sensor with MSRLV.

In the proposed scheme, any sensor that tries to report false location information cannot pass the scheme because it is not able to collect the necessary data. Figure 5 shows an example of how the scheme successfully verifies a false location report. In this example, a sensor *M* tries to spoof its position as if it is located at the position of fake sensor $M^{\prime }$. When the sensor *V* wants to send packets to the sensor *M* or suspects the location inconsistency of *M* through the technique mentioned earlier in this section, it starts the location verification process and checks its distance to *M* through D-filtering. Suppose that *M* passes the filtering by carefully locating its fake position. *V* then sends the LOC_PROOF_REQUEST to the claimant *M*. Upon receiving the request, *M* performs D-filtering to check whether there are malicious sensors among its neighbors. After filtering out the malicious neighbors, it calculates the MSR token between *M* and *V* based on the collected data. The necessary data can be collected during the usual sensing data exchange as in the left of Figure 5. The solid arrow indicates the data that are collected and used for the calculation of the MSR token. The dotted arrow is also data from neighbors, but the data will not be used for the calculation because they are outside the mutually-shared region of *V* and *M* ($MSR_{vm}$). Since *M* wants to fake its location at the position of $M^{\prime }$, the calculated value should result from common data between *V* and $M^{\prime }$. However, *M* is not able to know every data in MSR between $M^{\prime }$ and *V*, i.e., data from the sensor *H* in the right side of Figure 5, because it is not physically located at the position of $M^{\prime }$. This physical constraint forces *M* to use the common data that are originated from $MSR_{vm}$ for the location verification. Consequently, *M* cannot pass the location verification.

The proposed scheme works based on the following facts: there is a region called an effective MSR (EMSR) between two sensors if one of them tries to fake its location, and there is at least one sensor in the EMSR. The EMSR is shown as a gray shaded area, and it includes sensor *H* in Figure 5. The effectiveness of the proposed scheme will be analyzed in Section 5.1.2.

One consideration for the successful verification is that the MSR token used for the location verification should satisfy the properties that were explained in the previous section. In this paper, a random value appended to the data packet is used for the calculation of the MSR token. Together with the random value, the physical location of sensors guarantees the properties of the token. The suitability of the packets with a random value as the source of MSR token will be analyzed in Section 5. Note that it is possible to use any other factors instead of packets with a random value to derive the token.

### 4.4. Handling the Verification Failure of the Honest Sensors

**Figure 6:**
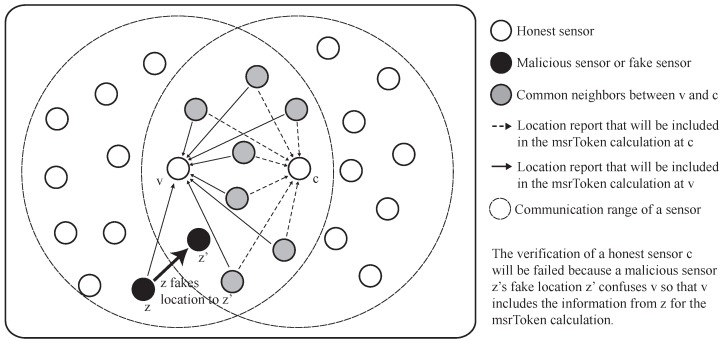
Verification failure example.

In this section, the purpose of D-filtering is described. If MSRLV does not perform D-filtering, the fake location report of any malicious sensor would cause an unintentional location verification failure of the honest sensor. When the malicious sensor who actually is located outside MSR between the honest verifier and claimant fakes its location at a position in the MSR, the verification for the honest claimant may not succeed. Figure 6 shows this case. In the example, assume that all of the sensors but *z* ($z^{\prime }$) are honest. When the verifier *v* tries to verify the location of the claimant *c*, *v* and *c* require the information from the shaded sensors in their $MSR_{vc}$, including black sensor $z^{\prime }$ for the successful verification. However, the information from $z^{\prime }$ cannot be acquired by *c* since the actual position of $z^{\prime }$ (i.e., *z*) is beyond the communication range of *c*. Thus, the information is not symmetric for *v* and *c*, and verification will fail. This situation may happen frequently and eventually degrade the successful verification.

Equipped with D-filtering, MSRLV can handle the case of the example in Figure 6. A sensor *v* will filter out the information from the sensor $z^{\prime }$ since the distance it measures through the filtering is different from its estimated distance through the reported location of $z^{\prime }$. Finally, the sensor *v* and sensor *c* will have the same msrToken value with the help of D-filtering.

From the perspective of the whole network, it is obvious that an attacker can influence the verification of the honest sensor if it passes D-filtering by conforming to the rule of the filtering. Those cases will be analyzed in Section 5.1.3.

## 5. Security and Performance Analysis

### 5.1. Security Analysis

This section discusses the security of the MSRLV. First, the purpose of the random value contained in the packet will be explained. Second, the security of the MSRLV will be shown by statistically analyzing theoretical attack situations. Third, the effect of D-filtering on the verification result will be discussed more carefully by considering the potential attackers’ behavior. Fourth, problems with sniffing tokens will be discussed. Finally, MSRLV’s responses to range-change, impersonation, wormhole and Sybil attacks will be discussed.

#### 5.1.1. Analysis of Using a Packet with a Random Value to Calculate the MSR Token

In this paper, the random value included in the data packet is used to calculate the MSR token. This packet, which contains a random value, will be shown to have all three of the required properties of a secure MSR token. The MSRLV operates on the assumption that there will be at least one packet exchanged between sensors within a certain time period. The rationale for this is that sensors in wireless sensor networks occasionally transmit the sensing data, such as temperature, pressure or wind speed, to the sink.

First, these packets have mutual symmetry since they can be observed by both the claimant and verifier in the model presented in this paper. Other sensors that do not locate at the right position are not able to listen to all of the necessary packets to calculate the MSR token. Even though malicious sensors are equipped with powerful devices, they can extend their transmission ranges only, not their listening ranges.

Second, the token derived from packets can be independently calculated by both the claimant and the verifier. Based on the assumption that the claimant and the verifier select the same packet to be used in the verification calculations, they do not need to exchange any other packets. Note that it is safe to assume that timing is synchronized between sensors because the data flowing through the sensor networks usually include the time, which the sensors use to calibrate themselves [24].

Finally, attackers are not able to deduce the token without knowing the data on which the calculation is based. The mathematical process to calculate the token is known to every sensor, but the data to calculate the token are available only to both sensors, since they come from the MSR between them. The physical constraint that the claimant must be at the correct location to collect data necessary to calculate the token makes it impossible for an attacker to know all of the packets generated from within the MSR. As shown in the Figure 5, there is an area (i.e., gray shaded in the figure) where the attackers cannot access. In addition, the fact that the piece of input data is a random value adds another layer of security against attackers from calculating the token.

#### 5.1.2. Analysis of the Successful Detection of Location Fraud

In order to successfully commit location fraud, an attacker has to fabricate the legitimate MSR token. If there is only one attacker, location fraud is not feasible because the attacker cannot collect all of the necessary packets to calculate the MSR token alone. The only way for the attacker to make a legitimate MSR token is by guessing the content of the packets that are transmitted from the MSR of the attacker and the verifier. However, it is also difficult to guess and fabricate the proper MSR token because we used the randomly-generated value to calculate the MSR token. Assuming that the attacker knows the verification process, the attacker can try to bypass D-filtering by carefully faking its location while still maintaining the same distance from the target verifier. This can be done by adjusting the angle between the line from the real location of the malicious sensor to the verifier and the line from the fake location of the malicious sensor to the verifier. We denote this angle as the fake-angle from now.

**Figure 7:**
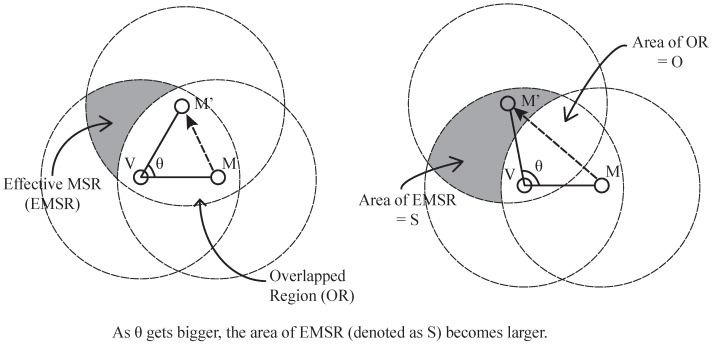
Analysis of the successful detection of a malicious sensor.

Figure 7 shows an example scenario in which a malicious sensor *M* tries to claim that it is located at $M^{\prime }$ by adjusting the fake-angle, *θ*. *M* must collect the packets from the MSR between *M* and *V*, especially including those originating in the EMSR. As a result, the successful detection of the malicious sensor can be achieved when there is at least one sensor in the EMSR. Then, we can expect the probability of successfully detecting a malicious sensor using simple statistics.

Since the deployment of sensors in the system follows a uniform distribution, the probability of no sensor being in the EMSR can be predicted. When we denote the entire network region for sensor deployment as *Z*, and its area as *A*, the probability distribution function (pdf) for any given sensor *i*’s coordinates (x, y) is as follows:

(3) $$f_{i}\left(x,y\right)=\left\{\begin{array}{cc} \frac{1}{A} & \;\mathrm{if}\;(x,y)\;\mathrm{is}\;\mathrm{within}\;Z\\ 0 & \;\;\mathrm{otherwise} \end{array}\right.$$

The probability of a sensor being located in the EMSR can then be calculated by multiplying the area of the EMSR, denoted as *S*, to the pdf Equation (3).

(4) $$P\left(i\;\mathrm{is}\;\mathrm{in}\;\mathrm{EMSR}\right)=S\times \frac{1}{A}$$

When there are *N* sensors in the network, the probability that no sensor is in the EMSR can then be calculated using Equation (4):

(5) $$P\left(\mathrm{no}\;\mathrm{sensor}\;\mathrm{in}\;\mathrm{EMSR}\right)=\Phi \left(S,A,N\right)={\left(1-\frac{S}{A}\right)}^{N}$$

As we can see from Equation (5), the probability denoted as the function Φ becomes smaller as *S* increases, assuming that *N* and *A* are fixed. In the other case, the function Φ decreases as *N* increases, assuming that *S* and *A* are fixed. Considering the applications of WSNs, *A* and *N* can be predetermined, leaving *S* as the only variable that is affected by the attacker’s fake location. When the fake angle grows, so does *S*, as shown in the Figure 7. Thus, an attacker’s chance of passing the verification process decreases by the proportion at which the faked location differs from the real location of the malicious sensor.

**Figure 8:**
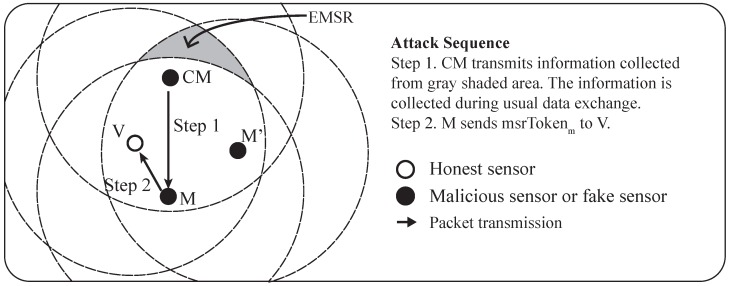
Colluding attack.

This analysis shows that an attacker *M* must cooperate with at least one other attacker, CM, which can overhear the packets sent from the EMSR, as shown in Figure 8. In this collaborative attack scenario, more than two attackers can collude to pass the location verification process. As shown in Figure 8, a malicious sensor *M* tries to fake its position as if it is at the position of virtual sensor $M^{\prime }$, and the colluding sensor CM assists *M* by delivering its neighbors’ packets, which are unreachable by *M*. Although the CM can gather the packets required for *M* to generate the MSR token, *M* should spend more time filtering out unnecessary packets after receiving all packets from CM. Thus, *M* will spend more time than the usual situation.

#### 5.1.3. Analysis on the Effect of D-filtering on the Verification Result

The malicious sensor can circumvent D-filtering by claiming a fake location that is the same distance from the other sensor, so sometimes D-filtering fails. D-filtering is performed to check the claimant’s location at the initial stage of the location verification process and to check the neighbors’ locations during the MSR token calculation stage of the process. In the initial stage of the protocol, if the malicious sensor bypasses the filter, it is expected that it will be caught later in the process. In this example, the verifier, *V*, is always an honest sensor, while the claimant, *C*, can be either an honest or a malicious sensor. The case where *V* is malicious is irrelevant because it would accomplish nothing by acting as such.

**Figure 9:**
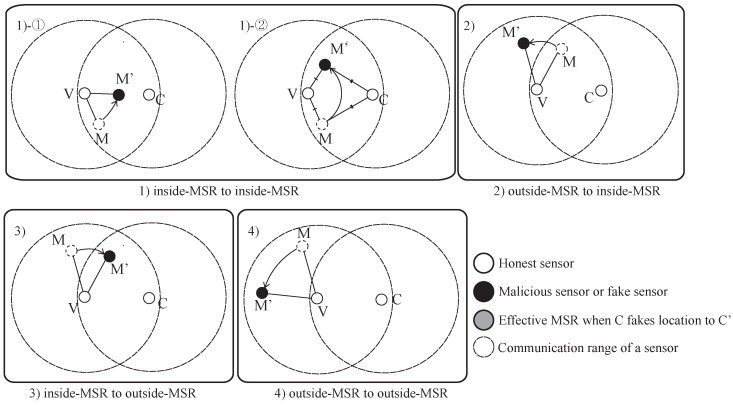
The effect of a malicious neighbor on D-filtering between honest sensors.

**Figure 10:**
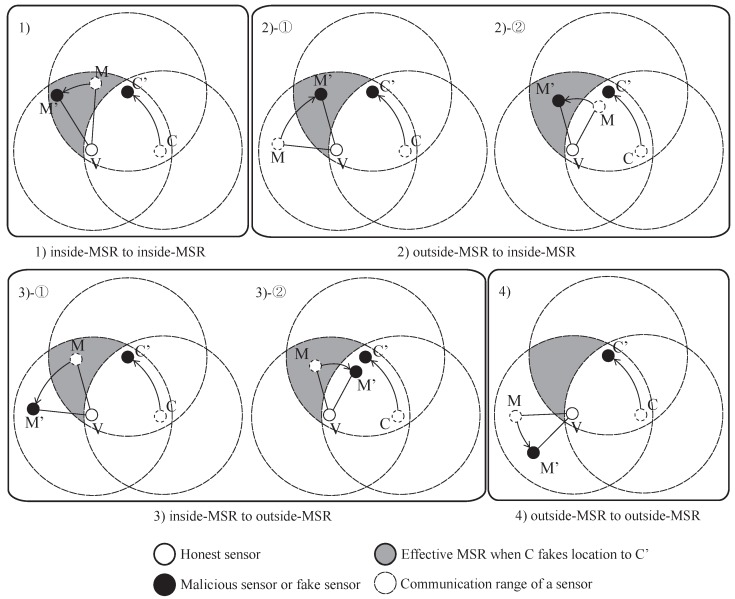
The effect of a malicious neighbor on D-filtering between honest and malicious sensor.

The result of the MSRLV is related to the faking behavior of the malicious sensor, *M*, which can be categorized as follows: (1) inside of the MSR, (2) from the outside to the inside of the MSR, (3) from the inside to the outside of the MSR or (4) outside of the MSR. Figure 9 and Figure 10 show all of the cases when the *C* is honest or malicious. For the simplicity of explanation, it is assumed that *V*, *C* and *M* are the only sensors in the network.

When *C* is honest, Figure 9 depicts what *M*’s faking behavior looks like. Case (1) has two possible outcomes: *M*’s fake location is consistent with the measured distance (1)-① to either *V* or *C*, or (1)-② to both of *V* and *C*. Cases (1)-① and (3) result in the verifier falsely rejecting *C*, while the Cases (1)-②, (2) and (4) have no effect on the outcome of the verification process.

In Cases (1)-① and (3), *M*’s data are included in *V*’s token, but are not included in *C*’s token. In Case (1)-①, *M* passes *V*’s D-filtering, but not *C*’s. In Case (3), *M* cannot even reach *C*. In each situation, *V* and *C* will have different MSR tokens, resulting in *C*’s false rejection.

In all other cases, *M*’s data are not incorporated into the token of *V* and *C*. Note that *C* does not use *M*’s data, because it is always outside of the MSR. In Case (1)-②, there are only two locations that have the same distance from both of the *V* and the *C* on a plane. Those locations are opposite each other, such that each location is inside or outside of the MSR. In Cases (2) and (4), *V* does not include *M*’s data in its token production since *M* lies outside of the MSR. Consequently, *V*’s token is not affected by *M*’s faking behavior, so verification is successful.

When *C* is malicious, the results of *M*’s faking behavior are different than when it is honest. Figure 10 shows each case of *M*’s possible faking behavior. Since the *C* fakes its location, Case (2) and (3) must be considered in detail. Each case has two sub-cases, as shown in Figure 10. Cases (1), (2)-①, (2)-② and (3)-② have no influence on the verification result, while Case (3)-① can result in the false acceptance of a malicious *C*.

In Case (1), *V*’s token is not affected because *M* will pass *V*’s D-filtering. *C* is outside of *M*’s communication range, so *C*’s token is also not affected. In Cases (2)-① and (2)-②, *M*’s data are accepted by *V* because *M* reported its location as being inside the MSR. *C* does not receive a packet from *M* or the packet fails to pass through *C*’s D-filtering process. In Case (3)-②, *V* will use the data from *M* because it is within the MSR, but *C* cannot because *M* is out of its communication range.

Case (3)-① results in the false acceptance case of a malicious sensor. As *M* fakes its location, *V* does not include its data in its token production process. Eventually, *V* and *C* will produce the same token.

#### 5.1.4. MSR Token Sniffing

**Figure 11:**
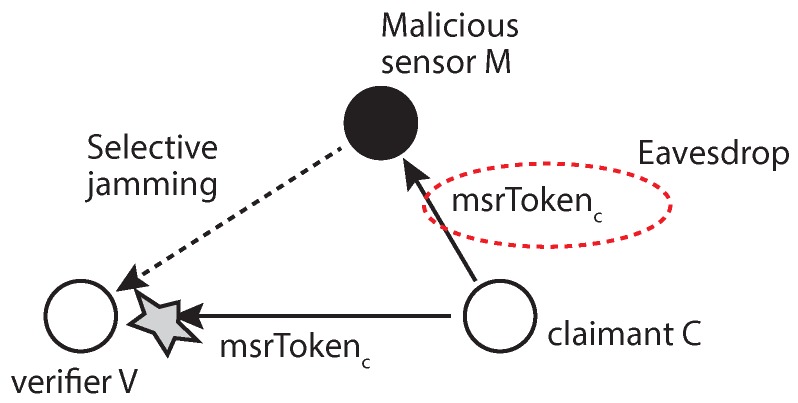
An example of sniffing on the msrToken.

The MSR token is calculated from a random value that is changed with every packet to inject randomness, freshness and, thus, security into the token production process. In order to pass the verification process by sniffing, the malicious sensor must perform the selective jamming attack [25] on the verifier to prevent it from accepting the claimant’s token, as shown in Figure 11. Without this jamming process, the verification process will complete while the malicious sensor sniffs the token. According to the attack model, the attacker can compromise a normal sensor to launch its attack in which case it is not easy to implement selective jamming with the resource-restricted sensor.

#### 5.1.5. Further Considerations on Known Attacks

Beyond some of the attack strategies discussed above, there are several others that warrant discussion. Attackers working alone to forge the token will be severely limited in gathering the necessary data. This is because the MSRLV works in a decentralized and node-centric way. Thus, attacks by multiple colluding attackers stand a better chance of success. Attackers may use some known attacks, such as range-change, impersonation, wormhole or Sybil attacks, to bypass the location verification. Even though those attacks may not be designed to attack the location verification process, it is worth mentioning the effect of those attacks on the location verification.

The range-change attack either shrinks or grows a sensor’s transmission range, though it does not affect its listening range. The reason for this is that the transmission power can simply be adjusted by a change in the device’s software, but increasing the reception ability requires additional hardware, such as a directional antenna. Thus, an attacker cannot forge the legitimate MSR token with this attack when its communication range does not cover the MSR between the verifier and its fake location. However, this attack can cause denial-of-service (DoS) because the change of transmission range can lead to the different token calculation at the verifier and the honest claimant. This is because the scheme is based on the assumption that the communication range of sensors is identical. The influence of the asymmetric communication range can be mitigated as discussed in Section 7.

Impersonation is an attack where one sensor pretends to be another sensor by sending incorrect location information with using the honest sensor’s identification information. This can cause DoS. However, this attack can be usually protected from through authentication. Even without authentication, attackers cannot gain much benefit through this attack when considering the following two cases. The first case is when an attacker in MSR impersonates the other sensor who is located outside the MSR. The second case is the opposite. In either case, D-filtering will mitigate the effect of the attack. Cases such as attackers in an MSR faking their location as the other location in the MSR and attackers outside an MSR faking their location outside the MSR are not effective attacks, so we did not consider them.

The wormhole attack tunnels the packets between colluding sensors. This attack is one kind of collaboration attack discussed earlier. If the malicious sensors use the out-of-band channel which is not affected by the radio signal of the target WSN, they can speed up the delivery of the necessary information from their neighbors. Currently, the scheme proposed in this paper is vulnerable to this kind of attack. However, it can be mitigated by adopting wormhole detection mechanisms already developed by other researchers.

A Sybil attack occurs when a malicious sensor represents multiple identities by creating a large number of pseudonymous identities. If the malicious sensor inside an MSR generates fake identities inside the MSR, there will be no problem because the verifier and the claimant can receive packets from the sensor, so the MSR token is not affected. However, a problem may occur in the other case, such that the attacker outside an MSR creates multiple identities inside the MSR. This attempt will affect the MSR token calculation. The verifier may revoke the verification because the tokens will not match. If the claimant is honest, there will be a DoS problem. Otherwise, the evil claimant will be detected.

Table 1 shows the considerations of some known attacks on our scheme, including other location verifications. MSRLV achieves reasonable defenses when compared with the other schemes. In the table, “Y” means the scheme can defend the corresponding attack, whereas “N” is not. “P” means possible to defend the attack if the location verification can be integrated with an appropriate technique or affected by the attack, but can mitigate its effect.

### 5.2. Performance Analysis

The performance of the proposed and related schemes was analyzed in terms of communication and computation costs when verifying a single sensor. This analysis is done based on each system model of those schemes. Communication cost was defined as the number of packets that must be exchanged during the location verification process. Packets exchanged to prepare the protocol, such as the initial distance measurement or neighbor determination, were not counted as part of the communication costs. This is because all of the relevant schemes in comparison require the update of neighbor information, and the update of the information can be done by exploiting the data packets delivering sensed data, such as temperature. In the same way, computation cost was defined as the computational complexity that a sensor needed to perform for the location verification process. Cryptographic operations were considered to have the same computation costs as basic operations, such as addition and subtraction, to give the maximum advantage to the other schemes. Note that our scheme does not perform cryptographic operations for the verification. The scheme proposed in this paper was compared to the phantom [14] and lightweight [15] on-spot verification schemes. Table 2 defines the terms used in the analysis, and Table 3 summarizes the communication and computation cost for each scheme.

Under the phantom method, each sensor measures its distance from each of its neighbors and then broadcast them. As packets used for protocol preparation were excluded from the communication cost in this analysis, the costs associated with this measurement are omitted. Thus, the final communication cost is the number of packets that are used to announce the measurements, which is the same as the number of the claimant’s neighbors. As such, the communication cost grows linearly according to the network’s sensor density. The computation cost is measured by $O(iter*N_{ne})$. It requires the encryption and decryption of the distance measurement information of all of its neighbors, which is a total of $N_{ne}+1$ cryptographic operations. $N_{ne}$ trilateration processes must be performed to filter out the malicious sensors during iter iterations. Here, iter is the number of trials that the algorithm must be run to successfully complete the verification process. Thus, the total computation cost can be represented as $iter*N_{ne}+\left(N_{ne}-1\right)$, which can be expressed as $O(iter*N_{ne})$ in complexity notation. In order to produce a location verification success rate of 99%, iter = 7 when 30% of the sensors are phantom nodes. The more compromised sensors there are, the more times the verification process must be run to maintain a particular success rate, so iter and the proportion of compromised sensors are positively correlated.

In the lightweight scheme [15], communication cost is N−1 because all of the sensors need to send their observation results to the central verifier. Here, neighbor observation packets are not counted. If only a single sensor malfunctions, it requires $\left(N_{ne}+1\right)$ communication costs since each sensor in the target area must send and receive its observation. In terms of computation cost, the lightweight scheme does not demand normal sensors to perform complicated calculations. Instead, the verifier alone performs all necessary verification calculations. The computation cost to the verifier then can be represented as O(N), since it has to perform computations for four matrices involving *N* sensors, all of which can be computed in the same loop.

By comparison to the previous two schemes, the MSRLV scheme communication cost is only two. Each verifier sends one verification request packet and receives one packet in response with the results of the process. In terms of computation cost, the verifier and the claimant should each perform XORs and distance calculations for their neighbors, so the total is $O\left(m\right)≃O\left(N_{ne}\right)$ in complexity notation.

**Figure 12:**
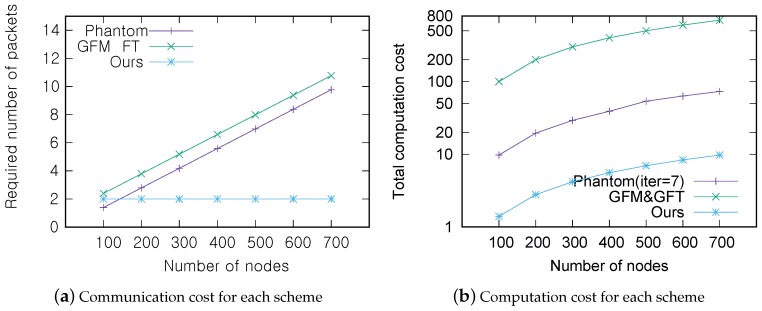
Cost for location verification of a single sensor: (**a**) communication cost for each scheme and (**b**) computation cost for each scheme.

Figure 12a,b show the communication and computation costs of each scheme according to the number of sensors in the network. For the comparison, it is further assumed that sensors are uniformly distributed in a 300 m × 300 m area and that the transmission range of a sensor is assumed to be 20 m. Thus, there are approximately 0–10 neighbors for any given sensor when there are a total of 100–750 sensors in the network. Figure 12b describes the result in log-scale because of the huge difference in the cost between methods. Note that the phantom scenario is depicted for the iter=7 case. When considering the case of moderate density among scenarios, i.e., 600 sensors in the network, the scheme proposed in this paper has a 77% better communication cost and a 92% better computational cost on average than both the lightweight and phantom schemes. Although the lightweight scheme cuts the computation cost of individual sensors down by employing the central verifier, its computation cost for the entire system is very high because the central verifier has to perform all of the computations. In light of these results, the proposed scheme is more effective at single sensor verification than competitive schemes.

### 5.3. Neighbor Table Management Overhead

For the MSRLV, a sensor in the network should manage the neighbor table in its memory. This could be an overhead when considering the limited memory of sensors. A neighbor table consists of the sensor ID, coordinates, a random value and RSSI. Each of the items takes up 2, 16, 8 and 8 bytes, respectively. Thus, total memory usage for maintaining one neighbor in the neighbor table is 34 bytes. Since the number of neighbors will be determined by the sensor density of the network, 0–10 sensors will be around a sensor when the total number of sensors in the network varies from 100–700. This leads to 0–340 bytes of memory consumption in each sensor. Considering the memory or storage capacities of sensors in the real world, the size of the neighbor table can be easily accommodated. Table 4 describes the supported memory size in each sensor product [26] and the required memory size for the location verification.

## 6. Performance Evaluation

The purpose of the evaluation is to verify the relationship between the variables proposed in Equation 5. The performance of the MSRLV was evaluated using the C_++_ language. The simulation results in this section, which are in accordance with the hypothesis presented in Section 5.1, can support the validity of our simulation.

**Figure 13:**
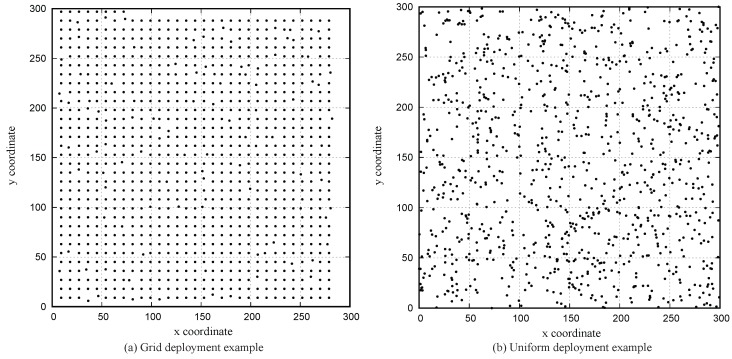
Deployment strategy of sensors in the target zone with 1000 sensors. Ten percent of them are smart attackers, and each attacker fakes its location by about 10 degrees.

As before, the sensors are deployed in a 300 m × 300 m square area. The position of sensors is determined based on the grid deployment strategy or the uniform random deployment. The grid deployment strategy is selected because the proposed verification is expected to perform the best when sensors are evenly deployed at a fixed distance. The test results under the grid deployment can be used to understand the performance under the uniform random deployment, which is a widely-used deployment strategy in the WSN. Each of the deployment strategy examples with some attackers is shown in Figure 13. Note that the evaluation in this paper is performed against two relatively stable deployment scenarios, such as grid and uniform, but it is expected to work in other deployment scenarios, such as a Gaussian distribution, if the network density is high enough. However, the proposed scheme may not work well when the network density becomes lower, i.e., at the edge of the network in the Gaussian deployment scenario. The communication range of sensors is 20 m based on the choices made in prior studies [11,15], though in the real world, the underlying standard of these sensors, IEEE 802.15.4, can support a range of up to 100 m.

The simulation does not consider the physical, MAC, routing layer functions of wireless sensor networks. If sensors can acquire the neighbor information reliably, any existing MAC and routing protocol can be used as the underlying protocol. In the simulation, the communication channels are assumed to be bidirectional between sensors. This means that sensors i and j can hear each other when they are within each other’s communication range. The asymmetric links are common in wireless networks and caused by physical issues, such as multipath fading or shadowing, but techniques, such as a handshaking, a proper coding scheme [27] or channel hopping [28], can be used to overcome the asymmetry of the links. By adopting those techniques, sensors can find and use the stable communication range while setting up the network, so that the sensors can gather the neighbor information reliably. However, one should note that the performance of the MSRLV will be decreased when the MSRLV is applied to the real-world application because the signal interference can be easily caused by obstacles in the real-world environment, and this cannot be completely eliminated by those mitigation methods.

The simulation was run according to the simulation parameters shown in Table 5. Each sensor in the network performed location verification on each of its neighbors. The total number of sensors in the network varied between 100 and 1000 by increments of 100, so that each sensor had its neighbors between zero and 13.

Malicious sensors accounted for 10% of the total. There were two types of attackers: smart and dumb. Dumb attackers claimed that they had a location chosen at random between 10 m and 20 m away from their real locations in any direction without considering which sensors would act as their verifiers. Smart attackers faked their locations by adjusting a fake-angle between 10 and 90 degrees, described in Section 5.1.2, in consideration of their target verifiers.

The purpose of the proposed location verification process is to decide whether the location of the claimant is acceptable, so its performance can be expressed by measuring its successful rejection rate (srr) and false rejection rate (frr). The srr is the percentage of malicious sensors that are properly filtered out, while the frr is the percentage of honest sensors that are improperly filtered out. Each rate can be described mathematically as follows:

$$srr=N_{fm}/N_{m}\;$$

where $N_{fm}$ is the number of malicious sensors filtered by the verification process and $N_{m}$ is the number of total malicious sensors participating in the verification process.

$$frr=N_{fh}/N_{h}\;$$

where $N_{fh}$ is the number of honest sensors with the correct location that is falsely filtered by the verification process and $N_{h}$ is the number of total honest sensors with the correct location that are participating in the verification process.

The total number of sensors and the fake-angle of the smart attacker were the main variables that influenced the srr. To show the influence of sensor density on the verification performance, we fixed the area of the target region for sensor deployment and vary the total number of sensors. In addition, to show the influence of the smart attackers’ behavior on the verification performance, we vary the fake-angle of the smart attacker.

Even though some other approaches adopting detection rate and false positives as a performance metric used the concept of the receiver’s operating curve (ROC) [19,20,21], the MSRLV does not use the ROC. This is because the false positive, frr in this paper, is affected by the behavior of the malicious sensors, which is not under our own control. The detailed explanation about this will be provided in Section 6.3.

### 6.1. Effect of Network Density

**Figure 14:**
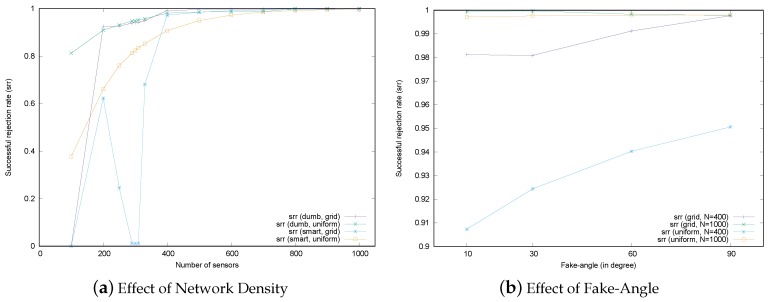
Successful rejection rate.

The location verification schemes considered in this paper make use of the information from neighbors, so the number of neighbors affects the verification performance. The number of neighbors for each sensor can be adjusted by controlling the network’s density, which is a combination of the total number of sensors and the distribution of sensor deployment in the field. Figure 14a shows the verification performance of the MSRLV in terms of network density. In the figure, the total number of sensors is displayed on the x-axis. On the y-axis, the percentage of the malicious sensors being successfully rejected by the MSRLV is displayed. Each line graph corresponds to the srr according to the combination of sensor deployment strategies and types of malicious sensors. The behavior of the attackers is set to either dumb or smart in each scenario. Smart attackers in this experiment fake their location by adjusting the angle by 10 degrees.

As shown in Figure 14a, verification performance generally improves as sensor density increases. This is because location verification depends on the implicit help of neighbors. Given that a sensor must be in the EMSR to correctly filter out malicious sensors, the more neighbors there are, the greater the chance that malicious sensors will be correctly filtered out. This result also accords with the relationship defined in Equation 5. Dumb attackers have a relatively high, stable srr across conditions, which indicates that D-filtering works well.

One interesting point is that the performance of the MSRLV significantly drops around 300 sensors in the network and recovers as the number of sensors increase. Under 300 sensors in the network, sensors are deployed 16 m away from each other. The majority of sensors in this situation do not have any neighbor in common, which makes the token value zero. This affects the performance because the scheme is designed to accept the same token as the successful verification, which means the sensor is classified as the honest one. Thus, the malicious sensors in this sparse network might be also classified as the honest ones. If this zero token is taken as the abnormal state of the sensor, the honest sensor will be considered as the malicious one, which leads to the increase of the successful and false rejection rate. The acceptance of this zero token can be determined by the policy of the service. However, as the number of sensors increases, any two sensors will have enough common neighbors, which results in the recovery of the successful rejection of malicious sensors.

The other point worth mentioning is the performance peak of the grid deployment scenario with the dumb attacker under 200 sensors. In this case, sensors are located 20 m away from each other. A malicious sensor’s attempt to fake its location toward a certain sensor will cause the situation that its location becomes out of communication range to the other sensors. This situation is interpreted as verification failure, so the srr increases.

In the case of the uniform random deployment scenarios, performance increases rapidly at the lower end of the density conditions because smart attackers have more physical empty space to fake in, as shown in Figure 13, though the benefits of this effect decrease exponentially as density increases linearly.

According to this result, it is obvious that the proposed scheme is not practical in the network with less than four neighbors. The network may become sparse because sensors can lose their function due to energy exhaustion or a harsh environment. However, it is reasonable to expect that the network would have more than five neighbors when considering usual applications of WSNs, such as machine diagnosis, vehicle tracking, smart homes or habitat monitoring [29].

### 6.2. Effect of the Fake-Angle of Malicious Sensors

The second factor that is related to verification performance is the fake-angle of the attacker. Figure 14b describes the srr of the MSRLV when the fake-angle is 10, 30, 60 and 90 degrees. For each fake-angle scenario, we set the total number of sensors to 400 and 1000 in the grid and the uniform random deployment strategy. The portion of the malicious sensor is fixed to 10% of the all of the sensors.

As expected, in the uniform random deployment, the srr is increasing in the sparse scenario with 400 sensors and almost maintained in the dense scenario with 1000 sensors. However, the srr remained constant throughout all grid deployment scenarios. This is because sensors are evenly distributed so that they can contribute to the verification.

### 6.3. Discussion on the False Rejection Rate

**Figure 15:**
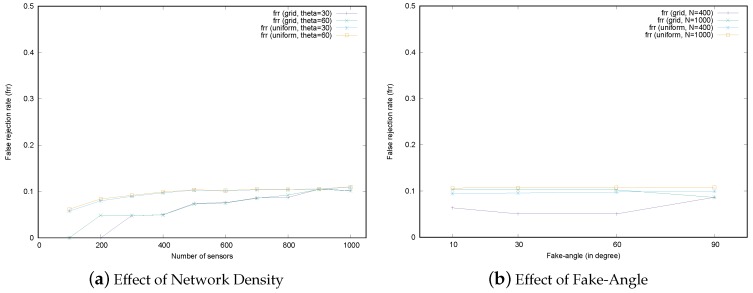
False rejection rates.

In the MSRLV, only smart attackers cause false rejections because they may cause honest sensors to fail to be verified as explained in Section 4.4. When there are only dumb attackers, the proposed D-filtering procedures filter out those kinds of malicious sensors at the first stage of the protocol. Thus, only the smart attacker scenarios are considered in this section. The frr according to the number of sensors and fake-angle under 10% of attackers is depicted in Figure 15. Even though this evaluation result does not show the false rejection rate for the honest sensors with no attackers around them, the MSRLV can suffer from packet loss in the real-world environment, which leads to the false rejection.

In the uniform random deployment scenarios, the frr is almost maintained the same as the portion of the attacker in the network. This result is related to D-filtering that may not successfully filter out the malicious sensors, as discussed in Section 5.1.3.

In the simulation, every honest sensor performed the verification on each of its neighbors. The false rejection occurs when a malicious sensor *M* fakes its location by targeting an honest sensor *A*, and the verification involves *A* as a verifier (or a claimant) and *M* as a neighbor. Since the proportion of that kind of verification is the same as the proportion of malicious sensors, the frr is almost maintained even though the number of sensors or fake-angle is changed.

In grid deployment scenarios, the frr increases very slightly as the density increases. In a relatively low-density network of 200–400 sensors, the frr was relatively lower than at other densities because the fake location of a malicious sensor does not fall into the MSR of the honest sensors around it. As the number of sensors increases, even a small fake in the location can be included in the MSR of honest sensors in the vicinity of the attacker.

In conclusion, the frr is affected by the amount of fake locations in the location of the malicious sensors whether they are the claimant or the neighbor of the verifier or claimant. Since the positions of the malicious sensors cannot be controlled by the system, the frr is not a controllable factor in the MSRLV. Fortunately, this result is not of any benefit to the attacker. However, the uncontrollable nature of the frr is the cost for achieving a higher srr.

## 7. Further Discussion

**Figure 16:**
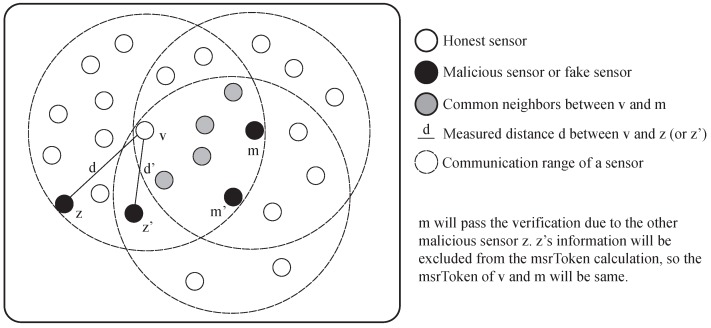
The side-effect of the distance-inconsistency filtering.

The distance-inconsistency filtering is helpful to reduce the verification failure of the honest sensors, but it has a side-effect. A malicious sensor can pass the verification unintentionally because of the other malicious sensors. Figure 16 shows the example of the consideration. When the malicious sensor *m* is trying to fake its location at the position of $m^{\prime }$, it needs the information from the MSR between *v* and $m^{\prime }$. At this moment, a sensor *z* also reports the false location of itself as if it is at the position $z^{\prime }$. Since the sensor $z^{\prime }$ is located in the most critical position for the successful verification of the sensor $m^{\prime }$, the information from the sensor $z^{\prime }$ should be included in the calculation of msrToken. However, the information from sensor $z^{\prime }$ will be excluded from the msrToken calculation since the estimated distance $d^{\prime }$ between sensor *v* and sensor $z^{\prime }$ is different from the measured distance *d* between sensor *v* and sensor *z*. Consequently, sensor *m* successfully passes the verification. However, the influence of the side-effect of the distance-inconsistency filtering is not huge under the moderate portion of malicious sensors, as shown in Section 6. This is because the design of the scheme is effective to suppress the impact of the side-effect when there are enough honest sensors in the network.

The next consideration is that the communication cost can be reduced more by using the piggybacking strategy. A verifier is able to send its location verification request in a piggybacked manner on the normal data packet, such as temperature data, to initiate the process. Thus, a claimant needs to send only one packet to finish the location verification process. Note that sensors cannot delay their response until the data packet is generated due to the collaboration attack case. In this case, the number of packets required can be decreased from two to one per verifier. Consequently, it would be possible to reduce the overall communication cost with this approach.

Third, the frequency of the location verification may be an important factor for the time-critical applications. The verification result can be cached for a certain period of time to reduce the verification overhead in the applications. However, it should be carefully designed because the prolonged validity of the verification result may give the adversaries a chance to bypass the verification process.

The fourth consideration is the vague communication range in the real world. Since the communication range is unclear in the real world, there is a chance that some neighbors receiving location verification requests with a weak signal power do not react to the location verification procedure correctly and degrade the performance of the scheme. This problem can be resolved by defining and using the range for the verification that is different from communication range. However, the difference of the verification range at each sensor will cause the malfunction of the verification. This makes the range fixed all of the time. In addition, the determination of the proper range would be a difficult problem. If the range is getting smaller, the area of EMSR becomes smaller, as well. This will cause a high probability of no sensor being in the EMSR, and it consequently leads to more occurrences of the false acceptance of malicious sensors. Thus, the range should be determined very carefully.

Finally, the mobility of sensors can be considered to widen the application of the proposed scheme. Basically, the proposed verification is designed for a static wireless sensor network. The mobility of any sensor will have the same effect as the malicious sensor. As a consequence, the honest sensors with mobility will experience false rejection, while the malicious ones will be successfully rejected in most cases. This is because the algorithm works based on the assumption that sensors always exchange packets for delivering sensing data to the sink. Thus, the mobility of a sensor will cause the lack of necessary information to calculate the correct msrToken at the verifier and claimant, even though they are honest ones. In order to support mobility, the algorithm should be changed to request the random value of the neighbors explicitly whenever the location verification occurs.

## 8. Conclusions

This paper provides an efficient location verification scheme by utilizing the MSR token. For this, we defined three properties of the MSR token: mutual symmetry, independent computation and anti-deduction. The mutual symmetry means that the same token should be acquired only by two participants, i.e., the claimant and verifier. The independent computation means that the participants can calculate the token without an explicit message exchange. The anti-deduction is that the token cannot be deduced by an attacker. For the source of the token that qualifies these properties, we have chosen the packets from the mutually-shared region between claimant and verifier. The analysis and evaluation substantiate that our scheme provides an efficient location verification in terms of communication cost, computation cost and verification performance. Furthermore, it would be possible to consider the effect of the piggybacking strategy, the side-effect of D-filtering, the frequency of location verification being invoked, the unclear communication range and the mobility, as mentioned in the Section 7, to make the verification process more effective. Those considerations may give a chance to improve the performance of the proposed scheme. We leave them for future work.

## Figures and Tables

**Table 1:** Consideration of defense against some known attacks. LAD, location anomaly detection; PLV, Probabilistic Location Verification; P, possible.

Scheme	Range-Change	Impersonation	Wormhole	Sybil
LAD [12]	P	P	P	P
PLV [13]	P	P	P	Y
Phantom [14]	Y	P	Y	Y
Lightweight [15]	P	P	N	P
Ours	P	Y	N	Y

**Table 2:** Terms and definitions.

Terms	Definition
*N*	Number of total sensors
$N_{ne}$	Average number of neighbors of a sensor, $N_{ne}<N$
*m*	Number of mutually-shared neighbors of two sensors, $m<N_{ne}$
iter	Iteration count for the speculative filtering (in the phantom scheme)

**Table 3:** Communication and computation cost for the verification of a single sensor.

Scheme	Communication Cost ($C_{comm}$)	Computation Cost ($C_{comp}$)
Phantom [14]	$N_{ne}$	$O(iter*N_{ne})$
Lightweight [15]	$N_{ne}+1$	O(N)
Our scheme (MSRLV)	2	$O\left(m\right)≃O\left(N_{ne}\right)$

**Table 4:** Memory size of each sensor product and memory consumption for the proposed scheme.

Sensor Product	RAM (KB)	Storage (KB)	Maximum Memory Consumption (B)
Btnode 3	64 + 180	4	340
mica2	4	512	340
mica2dot	4	512	340
micaz	4	512	340
telos A	2	256	340
tmote sky	10	1024	340
EYES	2	4	340

**Table 5:** Simulation setup and parameters for the proposed scheme.

Sensor Area	300 m × 300 m
Sensor deployment	Grid form/uniform distribution
Number of sensors	400/500/600/700/800/900/1000
Communication range	20 m
Portion of malicious sensor	10%
Attacker’s behavior (location fake)	Smart (10–90 degrees) / Dumb (10–20 m)
